# Differences in chemical constituents of *Artemisia annua* L from different geographical regions in China

**DOI:** 10.1371/journal.pone.0183047

**Published:** 2017-09-07

**Authors:** Xiaobo Zhang, Yuping Zhao, Lanping Guo, Zhidong Qiu, Luqi Huang, Xiaobo Qu

**Affiliations:** 1 College of Pharmacy, Changchun University of Chinese Medicine, Changchun, Jilin, China; 2 State Key Laboratory Breeding Base of Dao-di Herbs, National Resource Center for Chinese Materia Medical, China Academy of Chinese Medical Sciences, Beijing, China; 3 China Academy of Chinese Medical Sciences, Beijing, China; Tallinn University of Technology, ESTONIA

## Abstract

**Background:**

Daodi-herb is a part of Chinese culture, which has been naturally selected by traditional Chinese medicine clinical practice for many years. Sweet wormwood herb is a kind of Daodi-herb, and comes from *Artemisia annua* L. Artemisinin is a kind of effective antimalarial drug being extracted from *A*. *annua*. Because of artemisinin, Sweet wormwood herb earns a reputation. Based on the *Pharmacopoeia of the People's Republic of China* (PPRC), Sweet wormwood herb can be used to resolve summerheat-heat, and prevent malaria. Besides, it also has other medical efficacies. *A*. *annua*, a medicinal plant that is widely distributed in the world contains many kinds of chemical composition. Research has shown that compatibility of artemisinin, scopoletin, arteannuin B and arteannuic acid has antimalarial effect. Compatibility of scopoletin, arteannuin B and arteannuic acid is conducive to resolving summerheat-heat. Chemical constituents in *A*. *annua* vary significantly according to geographical locations. So, distribution of *A*. *annua* may play a key role in the characteristics of efficacy and chemical constituents of Sweet wormwood herb. It is of great significance to study this relationship.

**Objectives:**

We mainly analyzed the relationship between the chemical constituents (arteannuin B, artemisinin, artemisinic acid, and scopoletin) with special efficacy in *A*. *annua* that come from different provinces in china, and analyzed the relationship between chemical constituents and spatial distribution, in order to find out the relationship between efficacy, chemical constituents and distribution.

**Methods:**

A field survey was carried out to collect *A*. *annua* plant samples. A global positioning system (GPS) was used for obtaining geographical coordinates of sampling sites. Chemical constituents in *A*. *annua* were determined by liquid chromatography tandem an atmospheric pressure ionization-electrospray mass spectrometry. Relationship between chemical constituents including proportions, correlation analysis (CoA), principal component analysis (PCA) and cluster analysis (ClA) was displayed through Excel and R software version2.3.2(R), while the one between efficacy, chemical constituents and spatial distribution was presented through ArcGIS10.0, Excel and R software.

**Results:**

According to the results of CoA, arteannuin B content presented a strong positive correlation with artemisinic acid content (*p* = 0), and a strong negative correlation with artemisinin content (*p* = 0). Scopoletin content presented a strong positive correlation with artemisinin content (*p* = 0), and a strong negative correlation with artemisinic acid content (*p* = 0). According to the results of PCA, the first two principal components accounted for 81.57% of the total accumulation contribution rate. The contribution of the first principal component is about 45.12%, manly including arteannuin B and artemisinic acid. The contribution of the second principal component is 36.45% of the total, manly including artemisinin and scopoletin. According to the ClA by using the principal component scores, 19 provinces could be divided into two groups. In terms of provinces in group one, the proportions of artemisinin are all higher than 80%. Based on the results of PCA, ClA, percentages and scatter plot analysis, chemical types are defined as "QHYS type", "INT type" and "QHS type."

**Conclusion:**

As a conclusion, this paper shows the relationship between efficacy, chemical constituents and distribution. Sweet wormwood herb with high arteannuin B and artemisinic acid content, mainly distributes in northern China. Sweet wormwood herb with high artemisinin and scopoletin content has the medical function of preventing malaria, which mainly distributes in southern China. In this paper, it is proved that Sweet wormwood Daodi herb growing in particular geographic regions, has more significant therapeutical effect and higher chemical constituents compared with other same kind of CMM. And also, it has proved the old saying in China that Sweet wormwood Daodi herb which has been used to resolve summerheat-heat and prevent malaria, which distributed in central China. But in modern time, Daodi Sweet wormwood herb mainly has been used to extract artemisinin and prevent malaria, so the Daod-region has transferred to the southern China.

## Introduction

According to theory of traditional Chinese medicine, Daodi-herb acquires same unique characteristics[1], which has been naturally selected by traditional Chinese medicine clinical practice for many years. With higher quality, it comes from a particular geographic region, has more significant therapeutical effects and higher reputation other same kind of Chinese Medicinal Materials (CMM). According to the *Pharmacopoeia of the People's Republic of China* (PPRC),most kind of CMM has varieties of clinical efficacies, which is based on the chemical composition within the CMM. There is an obvious difference in the chemical composition of CMM in different areas. It is geographical locations of its growth that determines the different chemical composision of medicinal plants, hence presenting different efficacy.

As a kind of Daodi-herb, Sweet wormwood herb comes from the dried aerial parts of *Artemisia annua* L. Based on the PPRC, Sweet wormwood herb can be used to resolve summerheat-heat, prevent malaria, and relieve jaundices [2]. Since the Chinese scientist Tu Youyou discovered artemisinin as a promising antimalarial drug (for which she won the Nobel Prize in Physiology in 2015), there has been approximately 240 million people have benefited from the use of artemisinin, and an estimated 1.5 million people have escaped from death due to malaria [3]. Because of artemisinin, Sweet wormwood herb becomes a reputation for Chinese Medicinal Materials and Daodi-herb in China.

Plants within the same species show few differences in morphology, but they present significant variation in secondary metabolites based on their geographical locations. These variations are the result of genetic and environmental interaction, and a manifestation of biodiversity within a same plant species [4]. *A*. *annua* is a kind of medicinal plant that is widely distributed around the world and there are many kinds of chemical composition in it. Studies have shown that *A*. *annua* contains more than 100 types of chemical constituents [5], with a rich intra-species biodiversity in both proportion and abundance of secondary metabolites presented within the plant. Research has shown that scopoletin compatibility of artemisinin, arteannuin B and arteannuic acid also have antimalarial effective, and arteannuin B and arteannuic acid can be converted to artemisinin. Compatibility of scopoletin, arteannuin B and arteannuic acid have the medical functionof resolving summerheat-heat [6].

*A*. *annua* are widespread species, of which chemical constituents vary significantly according to geographical location. Studies have shown that *A*. *annua* is naturalized in many countries in Europe, northern Africa and North America [7]. Significant differences in the chemical constituents and their ratios extracted from *A*. *annua* samples collected from different regions have been reported [8, 9]. The distribution of *A*. *annua* may play a key role in the characteristics of its efficacy and chemical constituents. We therefore hope to study the relationship between those chemical constituents, (arteannuin B, artemisinin, artemisinic acid, and scopoletin) with special efficacy in *A*. *annua* growing in different provinces in china, and explore the relationship between chemical constituents and spatial distribution, in order to find out the relationship between efficacy, chemical constituents and distribution, and prove the unique characteristics of Daodi Sweet wormwood herb.

## Materials and methods

### Sample collection

A field survey was carried out to collect *A*. *annua* plant samples, 250 sampling sites were selected in 19 provinces in China, and 5 aerial parts of *A*. *annua* plant samples were collected in each sampling site, which were then dried under natural conditions. *A*. *annua* is one of the widespread species, distributed widely in various ecological environments. From July 2011 to August 2011, a total of 1,250 *A*. *annua* plant samples were obtained in 19 provinces in China, plant samples were no specific permissions were required for these locations. *A*. *annua* plant samples were identified by Dong Zhang from Institute of Chinese Materia Medica, China Academy of Chinese Medical Sciences. Geographical coordinates of those sampling sites were obtained by global positioning system (GPS).

### Chemical constituent determination

Chemical constituents in *A*. *annua* plant samples were determined by liquid chromatography tandem, an atmospheric pressure ionization-electro spray mass spectrometry. 5 μL of each sample was separated on an Agilent Zorbax SB-C_18_ column (150×2.1 mm, 5 μm) with an isocratic system acetonitrile–0.3% acetic acid water (60:40, v/v). The column temperature was 35°C and the flow rate was 0.3 mL/min. The optimized MS conditions for MS detector were as follows: capillary voltage, 3500 V; fragmentor voltage, 70 V; drying gas flow rate, 9.0 L/min; nebulizer pressure, 35 psig; drying gas temperature, 350°C. The selected ion monitoring (SIM) scanning mode was employed for quantification in negative and positive mode simultaneously. The detected protonated molecular ions were set at *m/z* 283, 249, and 193 respectively for artemisinin, arteannuin B, and scopoletin. However, as for artemisinic acid, the detected deprotonated molecular ions were set at *m/z* 233.

### Relationship analysis

The relationship between chemical constituents (arteannuin B, artemisinin, artemisinic acid, and scopoletin), was analyzed through Excel and R, a sophisticated open-source software for managing statistical calculations. Methods include correlation analysis (CoA), principal component analysis (PCA) and cluster analysis (ClA). The relationship between efficacy, chemical constituents and spatial distribution was presented through ArcGIS10.0, Excel and R software based on the geographical coordinates of 250 sampling sites, principal component and cluster analysis results.

## Results and discussion

### Relationship among chemical constituents in *A*. *annua*

According to the results of correlation analysis, there is a strong positive correlation between arteannuin B and artemisinic acid content (*p* = 0), while there is a strong negative correlation between arteannuin B and artemisinin content (*p* = 0). Correlation analysis results also show that there is a strong positive correlation between scopoletin and artemisinin content (*p* = 0), while there is a strong negative correlation between scopoletin and artemisinic acid content (*p* = 0). A more complete description of the relationship among chemical constituents is shown in Table 1. These results indicated that *A*. *annua* growing in the same region with higher artemisinin content has higher scopoletin content, while arteannuin B and artemisinic acid contents are lower.

**Table 1:** Correlation between chemical constituents in *A*. *annua*.

Chemical constituents	Arteannuin B	Artemisinin	Artemisinic acid	Scopoletin
Arteannuin B	1			
Artemisinin	−0.141**	1		
Artemisinic acid	0.785**	-	1	
Scopoletin	-	0.448*	0.133*	1

**: Correlation is significant at the 0.01 level

*: Correlation is significant at the 0.05 level.

**Fig 1:**
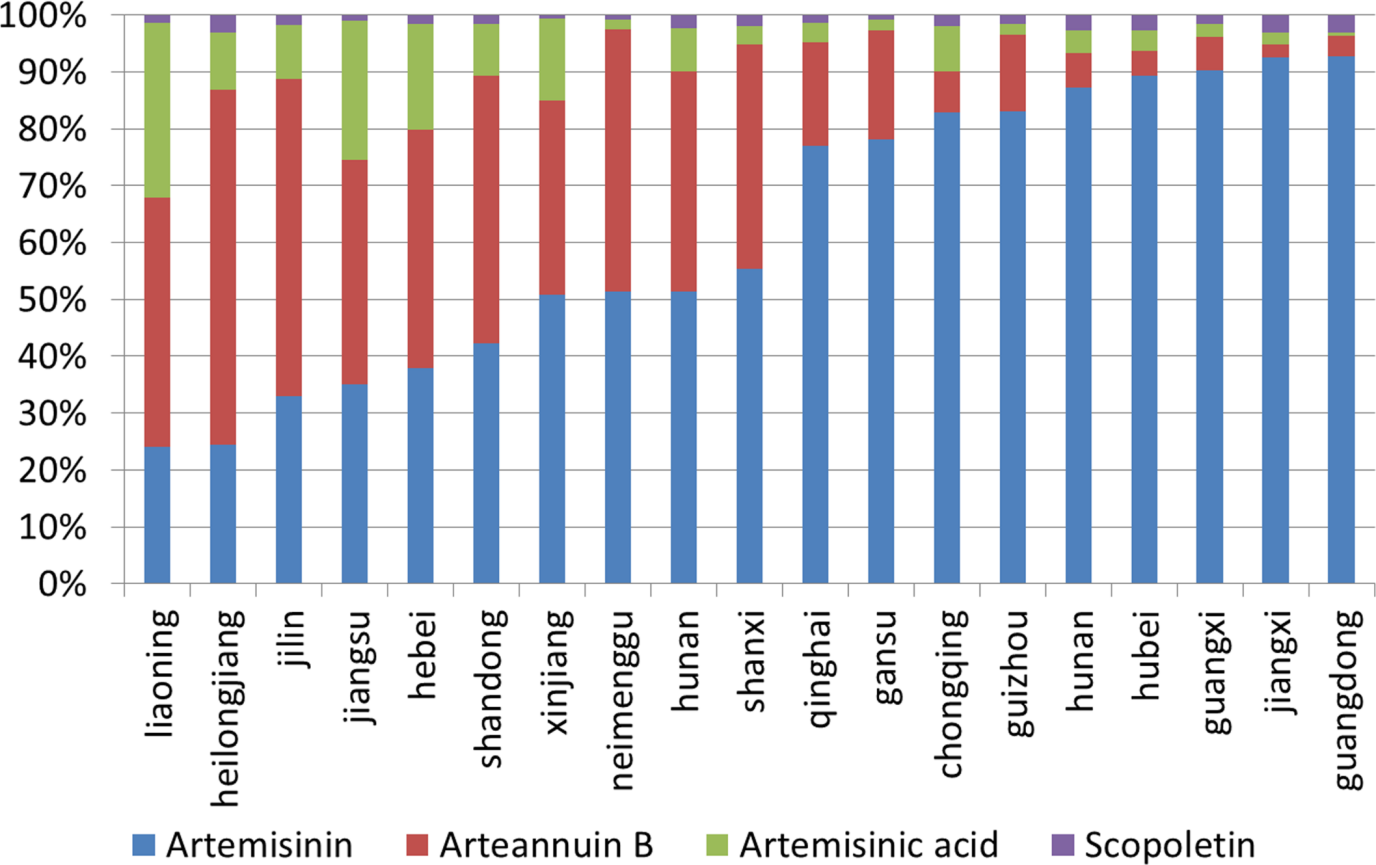
Proportions of chemical constituents in *A*. *annua*.

Based on the results of proportion analysis, there are big differences in proportions with the four chemical constituents in *A*. *annua*, and there are also large differences in *A*. *annua* growing in different provinces. A more complete description of the characteristics in the same one plant and in plants growing in different provinces is shown in Fig 1. So, the distribution of *A*. *annua* plays a key role in the characteristics of chemical constituents.

### Relationship between chemical constituents and efficacy

According to the results of principal component analysis, there were two principal component (PC) with eigenvalue greater than one and cumulative contribution rate greater than 80%. The first two principal components accounted for 81.57% of the accumulation contribution rate. The contribution of the first PC is about 45.12%, and the eigenvalue is 1.3433839. The contribution of the second PC is 36.45%, while the eigenvalue is 1.2075314. These results indicate that most of the characteristics in the chemical composition of the four chemical components were reflected in the two PCs. The main characteristics of the four chemical components can be expressed using the following two models:

$$Y_{1}=0.684\times X_{1}+0.703\times X_{2}+0.157\times X_{3}(Y_{1}:\;PC\;one,\;X_{1}:\;arteannuin\;B,\;X_{2}:\;artemisinic\;acid,\;X_{3}:\;scopoletin)$$

$$Y_{2}=0.158\times X_{1}-0.714\times X_{2}-0.682\times X_{3}\;(Y_{2}:\;PC\;two,\;X_{1}:\;arteannuin\;B,\;X_{2}:\;artemisinin,\;X_{3}:\;scopoletin)$$

The first model shows that the most influential variables on the variance of PC one were arteannuin B and artemisinic acid. Research has shown that compounds scopoletin, arteannuin B and arteannuic acid can be used to resolve summerheat-heat. Thus, the first PC can be applied as a measure to resolve summerheat-heat. It interpreted that Sweet wormwood herb’s summerheat-heat resolving efficacy is based on the first principal component, mainly due to arteannuin B and artemisinic acid.

The second model shows that the most influential variables on the variance of PC one were artemisinin and scopoletin. Research has shown that both artemisinin and scopoletin can be used to treat malaria. Thus, the second PC can be used as a measure to prevent malaria. It interpreted that Sweet wormwood herb’s malaria preventing efficacy is based on the second principal component, mainly because of the artemisinin and scopoletin.

### Relationship between chemical constituents and distribution

**Fig 2:**
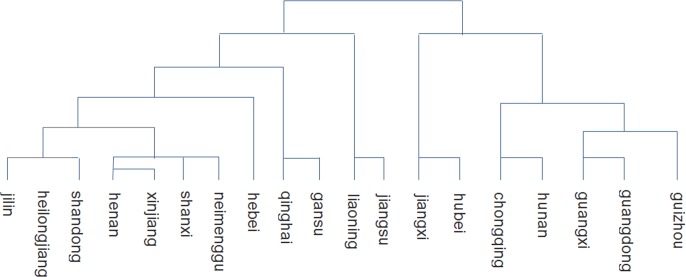
Clustering results of 19 provinces by principal scores.

The principal scores of the first two PCs were performed by standardized data, which is used to classify *A*. *annua* plant samples collected from 19 provinces could be classified into two groups based on cluster analysis. Group one include samples collected from seven provinces and cities including Jiangxi, Hubei, Chongqing Guangxi, Guangdong and Guizhou provinces. Group two include 12 provinces including Jilin, Helongjiang, Shandong, Henan, Xinjiang, Shanxi, Inner Mongolia, Hebei, Qinghai, Gansu, Liaoning and Jiangsu. A more complete description of the clustering results among provinces is shown in Fig 2. According to Fig 1 and Fig 2, the proportion of artemisinin in A. annua collected in provinces in group one is all higher than 80%.

**Fig 3:**
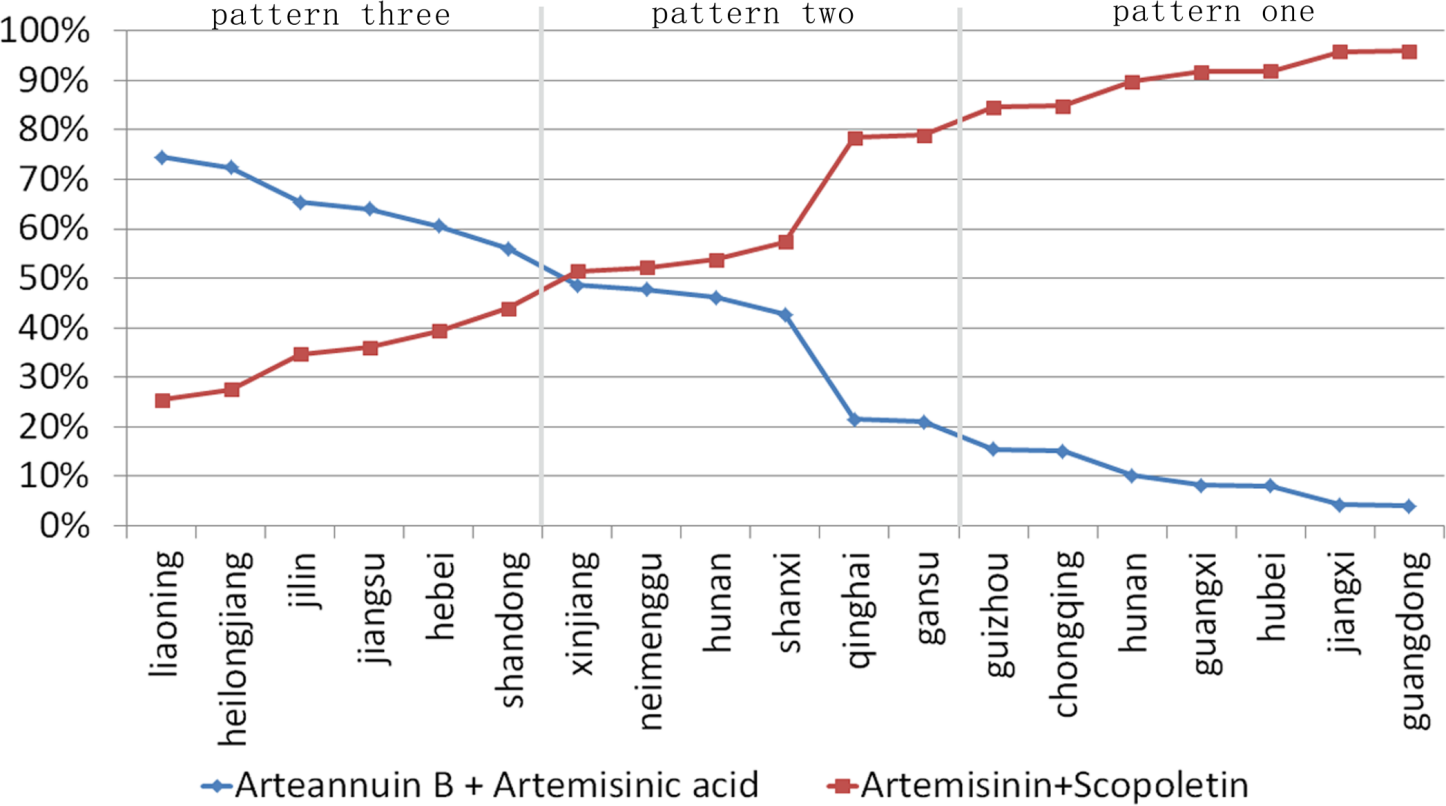
Proportions of two PCs in *A*. *annua* from 19 provinces.

In order to analyze the patterns of chemical constituent in *A*. *annua* samples collected from 19 different provinces, Excel was used to show the percentage of each principal component. The percentage of each PC’s main chemical constituents is shown in Fig 3. According to Fig 3, the proportion of the main compounds present in *A*. *annua* varies widely in samples collected in different provinces. According to the results of cluster and percentage analysis, samples could be divided into three groups based on their geographical locations, which means that there may have three chemical constituent patterns in *A*. *annua*.

Pattern one, the percentage of artemisinin and scopoletin in samples collected inseven provinces and cities including Jiangxi, Hubei, Chongqing Guangxi, Guangdong and Guizhou in group one are all greater than 80%, Pattern two, percentage of artemisinin and scopoletin in samples collected in six provinces in group two are all greater than 50% and less than 80%, including Jilin, Helongjiang, Shandong, Hebei, Liaoning and and Jiangsu. Pattern three, the percentage of artemisinin and scopoletin in samples collected in provinces in group two are all less than 50%, including Henan, Xinjiang, Shanxi, Inner Mongolia, Qinghai and Gansu.

**Fig 4:**
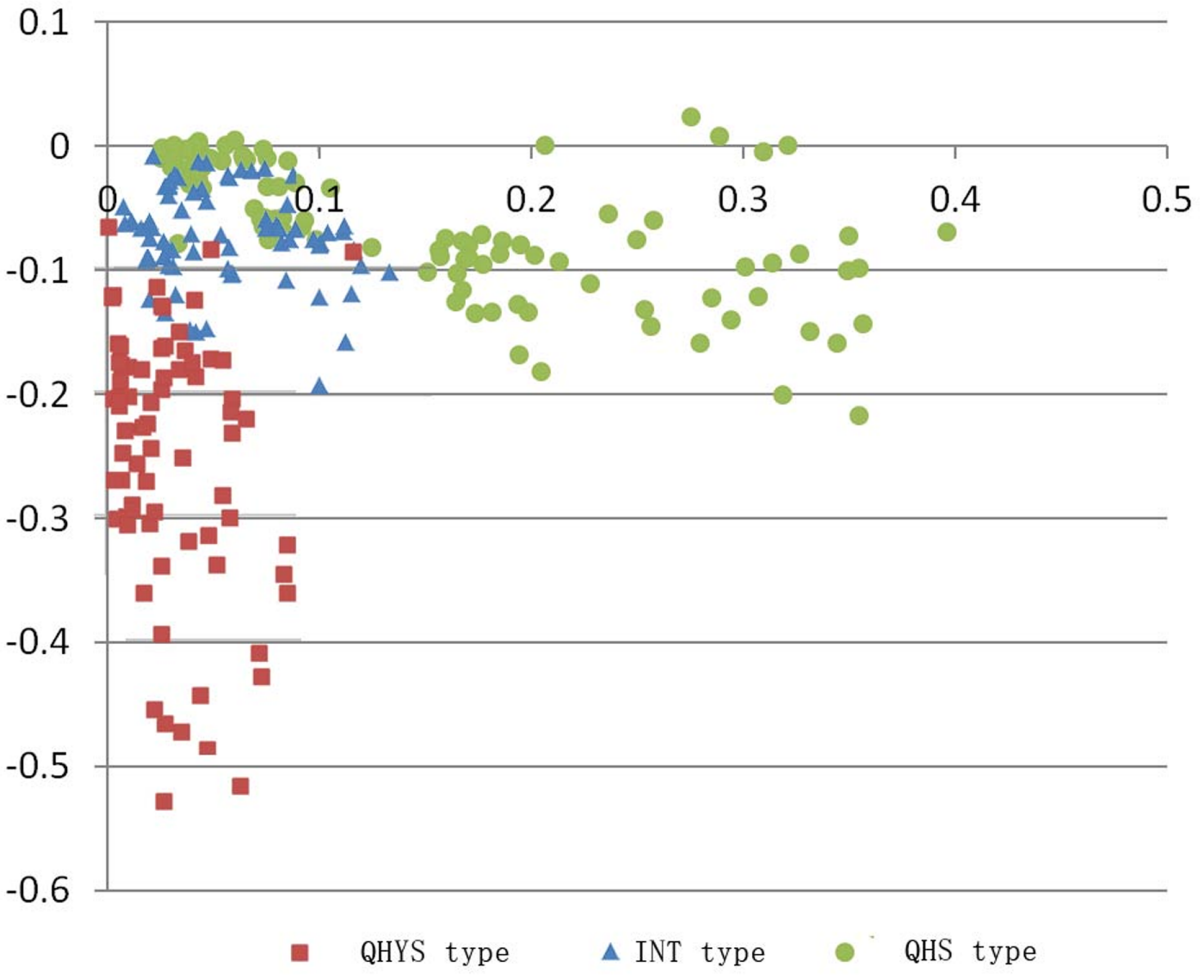
Scatter plot of the first and second PC's scores.

A scatter plot obtained by the first and second PC's scores using Excel, based on the 250 sampling sites from 19 provinces is shown in Fig 4. The results showed that the sampling sites from provinces in pattern one mainly distributed along the x-axis. The chemical type in *A*. *annua* of this group was defined as "QHYS type", of which the arteannuin B content is particular higher. The sampling sites from provinces of pattern two mainly distributed near the origin of scatter plot, with similar scores in both PCs. Chemical type in *A*. *annua* of this group was defined as "INT type", of which the artemisinin and arteannuin B contents are closer. The sampling sites from provinces of pattern three mainly distributed along the y-axis. The chemical type in *A*. *annua* of this group was defined as "QHS type", of which the artemisinin content is particular higher.

### Relationship among constituents, efficacy and distribution

According to the results of correlation analysis, there is a strong correlation of chemical constituent with its distribution and ecological environment factors. A more complete description of the relationship is shown in Table 2.

**Table 2:** Correlation between chemical components in *A*. *annua* and ecological factors.

Ecological factor	Arteannuin B	Artemisinic acid	Artemisinin	Scopoletin
Longitude (°E)	0.323**	0.285**	−0.239**	-
Latitude (°N)	0.147*	-	−0.681**	−0.513**
Altitude (m)	−0.387**	−0.323**	-	−0.176**
Radiation dose	-	.061*	−.145**	−.120**
Rainfall	−.113**	−.279**	.522**	.349**
Humidity	.059*	-	.646**	.383**
Temperature	−.078**	−.273**	.406**	.251**
Sunshine hours	−0.268**	0.082**	−0.156**	−0.085**
Annual frost-free period	−0.063*	0.651**	-	0.365**

**: Correlation is significant at the 0.01 level

*: Correlation is significant at the 0.05 level.

According to Table 2, there is a strong positive correlation between longitude, arteannuin B content (*p* = 0), and artemisinic acid content (*p* = 0), while there is a strong negative correlation between latitude, scopoletin content (*p* = 0), and artemisinin content (*p* = 0). The result of correlation analysis also shows that there is a strong negative correlation between altitude, arteannuin B content (*p* = 0), artemisinic acid content (*p* = 0), and scopoletin content (*p* = 0). Arteannuin B and artemisinic acid in the first PC presented a strong negative correlation with rainfall, temperature and altitude (*p* = 0). Scopoletin and artemisinin in the second PC, presented a strong positive correlation with rainfall, temperature and humidity (*p* = 0).

It can be interpreted that Sweet wormwood herb with excellent quality using to resolve summerheat-heat contents higher arteannuin B and artemisinic acid s, which mainly distributed in northern China, at temperate zones with lower altitudes. The Sweet wormwood herb with excellent quality using for preventing malaria has higher content of scopoletin and artemisinin, which mainly distributed in southern China, at subtropical zone with lower altitudes. A summary of the relationship between chemical constituents, efficacy and the distribution of Sweet wormwood herb is shown in Table 3.

**Table 3:** Relationship among chemical constituents, efficacy and distribution of Sweet wormwood herb.

Efficacy	Chemical constituents				Distribution		
	Chemical type	percentages (%)	PC1 (%)	PC2 (%)	Distribution	Topography (m)	Climate
mainly preventing malaria	QHS type	Artemisinin (>80) arteannuin B (<15)	>50	<50	South 31°N	<2000	Subtropical zone
resolve summerheat-heat or preventing malaria	INT type	Artemisinin (50–80) arteannuin B (15–50)	20–50	50–80	North 31°N, West China	500–200	Temperate zones
mainly resolve summerheat-heat	QHYS type	Artemisinin (<50) arteannuin B (40–60)	0–20	80–100	North 31°N, East China	<500	Temperate zones

According to the records from the *Collection of the Medical Records of the Palace of the Qing Dynasty*, Daodi Sweet wormwood herb (or high-quality Sweet wormwood herb) were obtained mainly from ancient Jingzhou (now the areas around Hubei province) [10]. According to the records from Ming Dynasty, Daodi Sweet wormwood herb was obtained mainly from ancient Jingzhou and Yuzhou (now areas around Hubei, Henan and Anhui provinces). *A*. *annua* covered in all the three chemical types can be found in these areas. The Sweet wormwood herb produced at these regions has been used to resolve summerheat-heat and cure malaria in ancient China. But in modern time, much more attentions are paid in efficacy of preventing malaria; thus Sweet wormwood herb are mainly used to extract artemisinin to treat malaria. Sweet wormwood herb with higher content of artemisinin is Daodi-herb. According to the previous studies and this paper, artemisinin content in Sweet wormwood herb growing in Chongqning, Guangxi and other southern provinces or cities is higher. So, the Daodi-region (or high-quality Sweet wormwood herb region) of Sweet wormwood herb has transferred to Chonqning, Guangxi and other southern provinces.

The harvest time period for *A*. *annua* is usually in the fall when flowers are in full bloom. Studies have indicated that arteannuin B and artemisinic acid are probable biochemical precursors to artemisinin [11]. Because of the shorter growth period of *A*. *annua* in northern China, large amounts of arteannuin B and artemisinic acid remain unconverted to artemisinin at the end of the summer, compared to the plants in the south that have higher content of artemisinin. In fact, it has been reported that if the growth period of *A*. *annua* in Heilongjiang province (at the extreme northeast of China) is extended, the content of artemisinin will increase [12]. It is suggested that *A*. *annua* from southern China could be used to resolve summerheat-heat, and arteannuin B could be extracted from the plant, through the harvest of *A*. *annua* earlier in the summer.

## Conclusions

In conclusion, this paper shows the relationship between distribution, efficacy and chemical constituents. The distribution of *A*. *annua* plays a key role in displaying characteristics of chemical constituents. In the same region, there are some *A*. *annua* has higher proportion of artemisinin and scopoletin, and lower arteannuin B and artemisinic acid contents, while there are others with higher arteannuin B and artemisinic acid content and lower artemisinin and scopoletin content. Thus, the first PC, distributing in northern China, can be used as a measure of resolving summerheat-heat. Such a medical efficacy is determined by the first principal component, mainly due to arteannuin B and artemisinic acid. The second PC, distributing in southern China, can be used as a measure to prevent malaria, which is determined by the second principal component, mainly because of artemisinin and scopoletin. This paper also proved the Chinese ancients saying that Daodi Sweet wormwood herb has been used to resolve summerheat-heat and preventing malaria, which was distribute in central China. But in modern time, the Daodi-region of Sweet wormwood herb has transferred to chongqing, guangxi and other southern provinces.

## Supporting information

S1 File Primary data for Figs. (XLS)
